# Malfunctioning sufentanil intrathecal pain pump: a case report

**DOI:** 10.1186/s13256-019-2314-2

**Published:** 2020-01-03

**Authors:** Lindsay Warner, Anna Branstad, Lindsay Hunter Guevara, Laura Matzke Bitterman, Matthew Pingree, Wayne Nicholson, Jason Eldrige

**Affiliations:** 1 Department of Anesthesiology, Mayo Clinic, 200 1st SW, Rochester, MN 55902 USA; 2 University of Wisconsin, Wisconsin, USA; 3 Department of Anesthesiology and Perioperative Medicine, Mayo Clinic, Rochester, MN USA; 4 Department of Pain Medicine, Mayo Clinic, Rochester, MN USA; 5 Department of Physical Medicine and Rehabilitation, Mayo Clinic, Rochester, MN USA

**Keywords:** Sufentanil, Chronic pain management, Pain pump, IDDS, Intrathecal opioid, Pain management

## Abstract

**Background:**

Sufentanil is a potent opioid uncommonly used to manage pain and is rarely administered via an intrathecal pain pump system.

**Case presentation:**

This case illustrates the use of intrathecal sufentanil in a 50-year-old Caucasian man for the management of chronic pain; however, the intrathecal drug delivery system experienced a malfunction which led to 1/100th output of the correct dosage. Interesting aspects of this case report include the uncommon choice of sufentanil use for an intrathecal drug delivery system, as well as the unusual pharmacokinetics of this drug. Specifically, this patient did not experience the major withdrawal that would be expected given significant under dosing of opioid, and this may be explained by the lipophilicity and context-sensitive half-times of sufentanil.

**Conclusions:**

Because of the absence of a clinically significant withdrawal in this case report, clinicians must be aware of relevant pharmacokinetic properties and unusual intrathecal drug delivery system technologies that influence a patient’s response when device malfunction occurs.

## Background

Intrathecal medications have been used for over 100 years with newer technology using reservoirs to delivery medication since 1981 [1]. To date, more than 300,000 intrathecal drug delivery systems (IDDSs) have been implanted for indications like discogenic pain, spinal stenosis, facet arthropathy, abdominal pain, complex regional pain syndrome (CRPS), postherpetic neuralgia, cancer pain, and unacceptable side effects from systemic opioids [2, 3]. Historically, morphine was the first spinal opioid used and is still very commonly used today [4]. Sufentanil is a newer opioid with 7.5 times the potency of fentanyl [5, 6].

Intrathecal sufentanil has been found to be effectively transferred into the circulatory system because of its lipophilicity and relative insolubility within the cerebrospinal fluid [7]. Similarly, total body sufentanil is known to accumulate over time due to sequestration within epidural fat due to its high lipophilicity [8]. In one animal model, sufentanil had limited free drug availability within the spinal cord, although it remained in the spinal cord tissues for a prolonged period of time [9]. With this concept in mind, there is concern that sufentanil could build up in the intrathecal fat and be redistributed back into the central nervous system (CNS) like a central compartment.

In this case, our patient was administered sufentanil through an implanted IDDS. He presented with an IDDS malfunction, which resulted in 1/100^th^ of the intended sufentanil dose being delivered. Surprisingly, no noteworthy opioid withdrawal was observed. Given the paucity of sufentanil use in the chronic pain medicine population, there is very little literature regarding the pharmacokinetics and clinical management of sufentanil in IDDS. Here we highlight the pharmacokinetic properties of sufentanil and some of the challenges of its use in intrathecal pain pump chronic pain management.

## Case presentation

Our patient was a 50-year-old Caucasian man, with a body mass index (BMI) of 31 and a past medication history significant for chronic back, neck, and leg pain since early 2000 after he sustained a traumatic fall at work. Other medical co-morbidities included degenerative joint disease, bursitis, depression, type II diabetes mellitus, hypothyroidism, testicular hypofunction, and hyperlipidemia. His family history and social history were noncontributory. The severity of his work injury ultimately required a cervical and lumbar fusion. The majority of his pain was localized to his low back and posterior legs without symptoms of CRPS. Three different types of pain were described: a constant and dull back pain, electric shocking pain in his legs, and numbness and tingling in his feet. After failing conservative opioid analgesic management, an intrathecal pain pump, Medtronic SynchroMed IIB, was placed at a non-Mayo facility in 2006 (10 years prior to malfunction). Records were not available detailing the reason for using sufentanil. After placement, his pain was significantly improved with an average daily numerical rating scale of 2–3/10. His pump was originally programmed with sufentanil (50 mcg/mL) with a daily dose of 38.307 mcg/day. Home medications included hydrocodone-acetaminophen 5 mg-325 mg (two tablets in the morning and two tablets in the evening) along with gabapentin 300 mg three times a day for neuropathic pain.

**Fig. 1:**
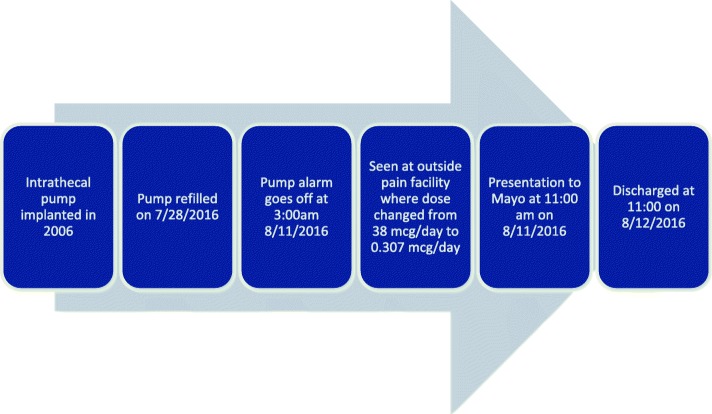
Timeline of case presentation events

Two weeks after his pump was refilled, he was awoken by the sound of an alarm signal from his pump. He presented to a local emergency department where the device was interrogated and found to have a rotor stall. Referral was made to Mayo Clinic for possible withdrawal management and pump refill. Exact timeline details are in Fig. 1. Prior to transfer, his dose was changed from 38.307 mcg/day to 0.307 mcg/day to lower the risk of a possible overdose in the setting of a malfunctioning pump. No additional opioid was given at that time. At the time that he presented to our institution, he rated his pain at 6/10 and denied any dizziness, nausea, sweating, diarrhea, or myalgias. A physican examination revealed a well-healed abdominal scar with some scar tissue thought to be related to prior wound dehiscence.

Interrogation of the pump revealed the lower dose of 0.307 mcg/day with an electric replacement indicator of 38 months. Drug was not being delivered despite an adequate reservoir volume, so the pump was deactivated and, again, it was thought to be related to a rotor stall malfunction. A fentanyl patch and patient-controlled analgesia (PCA) were provided for pain control and to prevent withdrawal symptoms. He reported no withdrawal feelings with stable vital signs that led the team to believe that his pump may not have been working for some time. After discharge, he had continued pain but elected to keep the pump in place.

## Discussion and conclusions

Sufentanil was used to manage this patient’s chronic pain via an IDDS. There are very few reports of sufentanil being used via continuous intrathecal infusion, with most of what is known of sufentanil intrathecal pharmacokinetics based upon single bolus dose administration [7, 10–13]. Opioids work by decreasing neurotransmitter release and hyperpolarizing membranes in the dorsal horn of the spinal cord. Drug is delivered to the mu receptor of the substantia gelatinosa, which is the ultimate site of action. Significantly less opioid is required when given intrathecally, compared to intravenous or oral doses, due to a large peripheral volume of distribution.

According to the manufacturer, these programmable IDDS devices deliver either an intermittent or continuous amount of medication intrathecally. Drug dosages can be changed without significant intervention such as the aspiration and refilling of a different medication concentration as seen in fixed-rate delivery systems. Programmable dose changes are quite useful for conditions such as opioid tolerance or dynamic changes in pain that necessitate frequent dose alterations for patients with cancer. The pump can be interrogated or deactivated without emptying the drug reservoir in cases of suspected malfunction [14].

The sufentanil IDDS used in this case is unique due to the degree of lipophilicity of sufentanil compared to other intrathecal opioids. Specifically, sufentanil has an octanol:water partition coefficient of 2842, compared to fentanyl’s coefficient of 717.0 and morphine’s of 0.7 [8]. The high lipophilicity of sufentanil probably contributed to significant sequestration of this drug in fat. The epidural fat acted as a depot for sufentanil, facilitating slow egress of the drug out of the fat and back into the systemic circulation, eventually reaching the CNS over time. According to the Polyanalgesic Consensus Conference recommendations, the recommended dose for sufentanil is 5–20 mcg with a maximum concentration of 5 mg/mL and maximum dose per day of 500 mcg [15].

The context-sensitive half-times of lipophilic opioids are also important in this case. Because of the pharmacokinetics of sufentanil, the stores of sufentanil in the body administered via continuous intrathecal infusion would be expected to change over time compared to that of single dose administration. For extended intravenously administered medications, the neural compartment target will saturate and redistribution to fat will occur. This would be difficult to assess by plasma levels because the majority of the accumulation is occurring in the fat stores making withdrawal dosing calculations extremely difficult.

Because the patient presented in this case report was receiving 1/100th of the dose prescribed, a major withdrawal would be expected. However, the contributory pharmacokinetics was such that this expected withdrawal was not observed. A dye study could have been performed to assess whether any medication was delivered; however, since our patient was not symptomatic and had decided to not continue with therapy, no additional root cause analysis was obtained. The pharmacokinetics of sufentanil may allow accumulation within adipose compartments due to its high octanol:water partition coefficient in comparison to other opioids (for example, fentanyl or morphine) and, consequently, the intrathecal delivery may favor epidural fat accumulation. Although, notably, accumulation in the peripheral sites within the central compartment would be less when compared to an intravenous route [8]. The absence of any major withdrawal is important because the specific drug pharmacokinetics appeared to work in this patient’s favor. Drug delivery system failure must include a broad differential that includes malfunction of both the delivery system and possibly the pharmacokinetics in order to safely deliver care to these patients. A recommendation was made for pump replacement and pain rehabilitation; however, our patient did not pursue either option. No cause was identified for the pump malfunction since the pump still remains in place several years later. The role that his home medications (opioids and gabapentin) played in his withdrawal symptoms cannot be excluded.

## Data Availability

Not applicable.
